# **Effect of variation in miRNA-binding site (rs8176318) of the **BRCA1** gene in breast cancer patients**

**DOI:** 10.3906/sag-1905-17

**Published:** 2019-10-24

**Authors:** Mushtaq AHMAD, Fazal JALIL, Mutiul HAQ, Aftab Ali SHAH

**Affiliations:** 1 Department of Biotechnology, Faculty of Biological Sciences University of Malakand, Chakdara Pakistan; 2 Department of Biotechnology, Abdul Wali Khan University Mardan (AWKUM) Pakistan; 3 Institute of Radiotherapy and Nuclear Medicine (IRNUM), Peshawar Pakistan

**Keywords:** ****Case-control study, single nucleotide polymorphism, untranslated regions, miRNAs

## Abstract

**Background/aim:**

A variation in the 3 prime untranslated regions (3′-UTRs) affects the binding of microRNA (miRNA) to the breast cancer (BC) susceptibility gene 1 (*BRCA1*) gene, and thus regulate its expression. In this study, the consequences of a variation in the miRNA-binding site (rs8176318G>T) in the 3′-UTRs of BRCA1 and its association with the risk of BC were investigated.

**Materials and methods:**

The selected variation (rs8176318G>T) was genotyped in BC patients (n = 300) and healthy controls (n = 300) using allele-specific polymerase chain reaction (PCR) [tetra-primer amplification refractory mutation system-PCR (T-ARMS-PCR)]. The results of the T-ARMS-PCR were further confirmed by Sanger sequencing through a random selection of 10% previously analyzed samples by T-ARMS-PCR. Association of this variation with BC was tested by calculating the odds ratio (OR) (at 95% CI) and χ2-value using 4 different genetic models (codominant, dominant, recessive, and additive models).

**Results:**

Using Fisher’s exact test, a significant association between variant rs8176318 (G>T) and BC was found in codominant [χ2-value = 15.68, df: 2 P < 0.0004], dominant [OR = 1.557 (1.082–2.241), P <0.0213], recessive [OR = 0.474 (0.3204–0.7017), P = 0.0002] and additive models [OR = 1.609 (1.282–2.018), P < 0.0001].

**Conclusion:**

It was therefore concluded that there is a significant association between rs8176318 and BC risk in a case-control study in a Pakistani population. Furthermore, an association study using a large sample size is required to further verify these findings.

## 1. Introduction

Breast cancer (BC) is estimated to be the second leading cause of cancer related death in women worldwide [1]. There are several environmental and genetic factors contributing to BC. Among genetic factors, a number of protein coding and noncoding genes contribute to the etiology of BC. BC susceptibility gene 1 (*BRCA 1*)**is one of the main protein-coding genes related to the causes of BC. Mutations in *BRCA1 *account for about 40%–45% of BCs [2]. Defects in *BRCA1* affect the cell cycle at various points, destabilizing the DNA and triggering DNA damage response, hence increasing the formation of tumors [3,4]. *BRCA1* is specifically involved in repairing breaks in double-stranded DNA, transcription, and recombination. The frequency and types of mutations in BRCA1 vary among different populations worldwide. The 3 prime untranslated regions (3′-UTRs) of *BRCA1* play a pivotal role in the stability, localization, and transport of mRNA, hence affecting its expression [5,6]. Single nucleotide polymorphisms (SNPs) in the 3′-UTRs affect the expression of genes, thus influencing the risk of cancer development. It has been reported that SNPs in the 3′-UTRs of *BRCA1* disturb the binding sites of microRNA (miRNA). Therefore, it can be used as a biomarker for BC [7]. A previous study reported that the homozygous (TT)/heterozygous (TG) variant is linked with increased risk of BC [8].

MiRNAs are small noncoding RNA molecules that control gene expression by regulating their corresponding mRNA. They act by binding to their target through miRNA:mRNA complementary base pairing at the untranslated regions of the genes. The capability of miRNAs binding to their target mRNA in the 3′-UTR is crucial for the proper expression of proteins. However, the binding capacity can be adversely affected by mutations/SNPs in the sites to which miRNA binds in the gene [9]. 

These observations lead to the assumption that unidentified variations in the miRNA binding sites at the 3′-UTR of the *BRCA1* could provide an important insight into the etiology of BC risk factors. 

Several techniques are used for the detection of mutations in the genome. However, tetra-primer amplification refractory mutation system-polymerase chain reaction (T-ARMS PCR) is a flexible, fast, and inexpensive SNP discovery strategy when compared to other genotyping tools [10]. It includes a single PCR and subsequent gel electrophoresis [11]. It utilizes 4 primers, 2 inner and 2 outer. The 2 inner primers are allele-specific and result in allele-specific amplification. The inner primer amplification depends on the genotype used in the PCR. The 2 outer primers act as an internal control and generate the outer fragment of the flanking locus of the SNP. Unequal positioning of the inner primers from the corresponding outer primer results in the generation of PCR products with diverse sizes, which are then easily identified in the gel [12].

In the current study, it was sought to investigate SNPs in the 3′-UTR of the *BRCA1* and explore their association with BC in sporadic BC patients.

## Materials and methods

### 2.1. Study subjects and ethical approval

This study included female patients (n = 300), ranging in age from 22 to 68 years, with confirmed BC at the Institute of Radiotherapy and Nuclear Medicine, in Peshawar, Pakistan, and matching controls (n = 300), ranging in age from 20 to 70 years, with no history of cancer, as shown in Table 1. 

**Table 1:** Categorization of the preclinical data for each patient with their age and cancer. stages (l–lV).

Age group	Stage 1	Stage II	Stage III	Stage IV	Total
20s	3	9	3	6	21
30s	7	27	21	12	67
40s	7	34	22	16	79
50s	3	31	40	13	87
60s	5	8	19	5	37
70s	0	0	9	0	9
80s	0	0	0	0	0
Grand total	25	109	114	52	300

Informed consent was given by all patients and healthy controls included in the present study. The present study followed the guidelines of the Helsinki declaration. The current research work was approved by the Advanced Study and Research Board at its 40th meeting held on March 29, 2017, University of Malakand, Pakistan.

### 2.2. Inclusion/exclusion criteria and genomic DNA extraction

The inclusion criterion for this study was a confirmed history of BC, while women with no history of BC were excluded. A total of 5 mL whole blood was collected from the BC patients and healthy controls in EDTA tubes and stored at refrigeration temperature until further processing. Genomic DNA was isolated from the collected blood samples using the phenol-chloroform method and it was stored at –20 °C until further processing.

### 2.3. PCR-based amplification and Sanger sequencing of BRCA1 gene

To amplify the 3′-UTRs of the *BRCA1*, 2 sets of primers (Table 2) were used under the following thermocycling conditions: initial denaturation at 95 °C for 10 min, followed by 35 cycles of 95 °C for 1 min, primer annealing at 56 °C for 30 s, extension at 72 °C for 1 min, and final single step extension at 72 °C for 10 min. The PCR product was visualized on 1% agarose gel by staining with ethidium bromide.

**Table 2:** List of primers used for amplification of the selected region of the BRCA1.

BRCA 1 primers	Primer sequences	Product size
BRCA1 3′-UTR 1 forward	5-AGCACTCTACCAGTGCCAG-3	646 bp
BRCA1 3′-UTR 1 reverse	5-AGGTTTCAAGTTTCCTTTTCA-3	
BRCA1 3′-UTR 2 forward	5-GAGTGCTTGGGATCGATTATGTGACTT-3	678 bp
BRCA1 3′-UTR 2 reverse	5-GCAACAGCTTCCTTCCTGGTGGG-3	

The amplified region was commercially sequenced using the Sanger sequencing method and the sequencing results were compared with the reference sequence obtained from the National Center for Biotechnology Information (NCBI). The analysis was carried out using Vector NTI software. Single nucleotide variants were detected.

### 2.4. Allele-specific T-ARMS-PCR amplification

**Figure 1:**
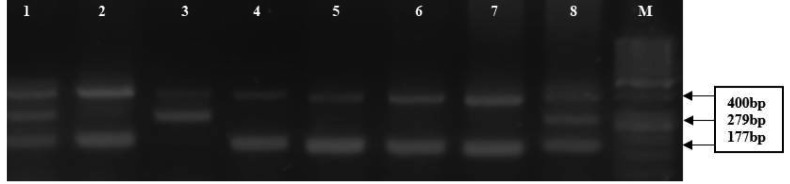
Gel electrophoresis image of the T-ARMS-PCR for the detection of polymorphisms in the BRCA1. Genotypes are indicated (lanes 1 and 8: GT; lane 3: TT; lanes 2, 4, 5, 6, 7: GG genotypes and M: marker).

The SNP (rs8176318; G>T) in the 3′-UTRs of the *BRAC1* was identified through Sanger sequencing. Further screening was performed for genotyping of the selected SNPs in the patients and controls using the T-ARMS PCR technique. Primer 1 software, available online (), was used to design allele-specific primers for the identified SNPs (Table 3). A PCR reaction mixture of 30 µL in a 0.2-mL PCR tube containing approximately 100 ng/µL of DNA template, 17 µL of green-Taq PCR master mix (Thermo Fisher Scientific Inc., Waltham, MA, USA), 4 µL of primers (1 µL of forward inner, 1 µL of inner reverse, 1 µL of forward outer, and 1 µL of reverse outer primer) (each primer of 10 pmol/µL), and up to 7 µL of nuclease-free water was prepared and processed. The amplification conditions used were initial denaturation at 95 °C for 10 min, followed by 35 cycles of denaturation, annealing, and extension at 95 °C for 1 min, 56 °C for 40 s, and 72 °C for 40 s, respectively. This was followed by the final extension step at 72 °C for 10 min. The products of amplification were separated by subjection to electrophoresis on 3% Agarose gel. Ethidium bromide was used for the staining and visualization of the PCR products. The results were confirmed by reproducing 15% of the samples and the accuracy of reproducibility was 99%. Furthermore, the results of the Sanger sequencing and T-ARMS PCR were 100% reproducible. The 3′-UTRs of the* BRCA1* were wild (GG) as well as mutant (TT) genotypes, which yielded 177 bp, 279 bp products, while the outer primer product was 400 bp, respectively, as depicted in Figure 1.

### 2.5. Statistical analysis

The Hardy–Weinberg equilibrium was applied as quality control to check the observed and expected genotype frequencies in both the patients and controls. Codominant, dominant, recessive, and additive models were applied on the collected data. The odds ratio (OR) at 95% confidence intervals (CIs) was also calculated. P < 0.05 was considered significant.

## 3. Results

**Figure 2:**
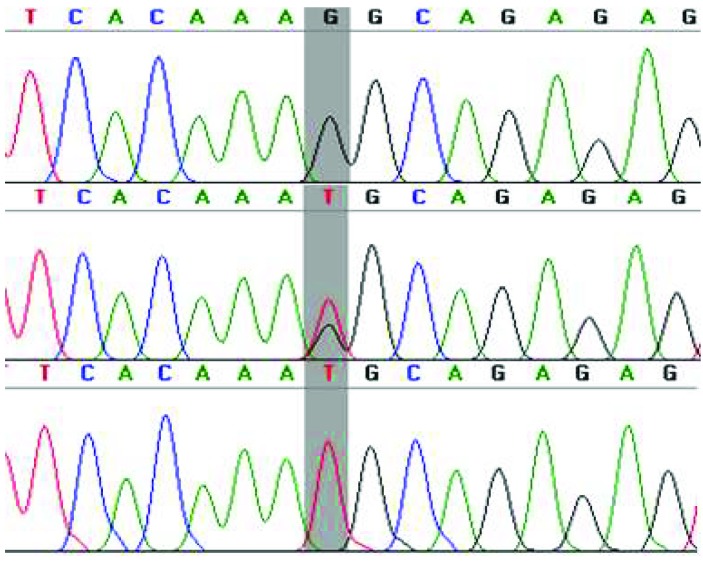
Validation assay to confirm the T-ARMS-PCR result using sequencing. The sequencing result of rs8176318 showed homozygote (GG), heterozygous, and (CG) genotypes using reverse primer, respectively.

Through the alignment of the DNA sequences of the *BRCA1* against reference sequences from NCBI, using gene sequencing analysis software, a genetic variant (rs8176318; G>T) in the 3′UTRs of the BRCA1 was identified. The variant was presented as homozygous (GG), homozygous (TT), and heterozygous (GT) in the analyzed samples (Figure 1). Furthermore, genotype data obtained through the T-ARMS PCR were statistically analyzed for association with BC (Table 4). Selected samples were sequenced to confirm the result of the T-ARMS PCR. The results obtained by the T-ARMS PCR were concordant with the sequencing (Figure 2). The distribution of genotypes was found to be consistent with the Hardy–Weinberg equilibrium in both the BC patients (P = 0.5707) and the controls (P = 0.4672). In the codominant model (genotype frequency distribution), frequency of the GG genotype was 31.33% (n = 94) in the BC patients and 22.67% (n = 68) in the controls. Similarly, frequency of the GT genotype was higher in the BC patients (52.0%, n = 156) than in the controls (47.67%, n = 143), whereas the frequency of the TT genotype was significantly lower in the BC patients (16.67%, n = 50) than in the controls (29.67%, n = 89). Thus, a significant statistical difference in the genotypes was determined through the codominant model analysis (χ2= 15.68; P < 0.004), indicating the role of this variant in the risk of BC.

However, through the additive model (allele frequency distribution) analysis, it was found that the G allele was significantly higher in BC patients (57.33%) than in the controls (45.51%). Thus, a significant difference in the allele frequency was determined through the additive model analysis in BC patients when compared to the controls, with the following 95% CI: OR = 1.609 (1.282–2.018); P < 0.0001, indicating that the G allele was a susceptible allele, while the T allele was protective. Similarly, both the dominant (GG vs. GT + TT) and recessive (TT vs. GT + GG) statistical models showed a significant association with the risk of BC, with a 95% CI of P < 0.0213 and P < 0.0002, respectively, indicating that individuals with the GG genotype were at risk of BC, while those with the TT genotype were protected from this risk. 

**Table 4:** Genotype and allele frequencies between the subject and control groups at locus rs8176318) in the miRNA-binding site of the BRCA1 using the codominant, dominant, recessive, and additive statistical models.

Statistical models	Genotypes	Patients (age%)	Controls (age%)	Fisher’s exact testOR (95% Cl)	χ2 -value,df	P-value
Codominant	GGGTTT	94 (31.33)156 (52.0)50 (16.67)	68 (22.67)143 (47.67)89 (29.67)	-	15.68, 2	<0.0004
Dominant	GG TT + GT	94 (31.33)206 (68.67)	68 (22.67)232 (77.33)	1.557(1.082–2.241)	_	<0.0213
Recessive	TTGG + GT	50 (16.67) 250 (83.33)	89 (29.67)211 (70.33)	0.4742(0.3204–0.7017)	_	<0.0002
Additive	GT	344 (57.33)256 (42.67)	279 (45.51)334 (54.49)	1.609(1.282–2.018)	_	<0.0001

## 4. Discussion

In the current study, the association of (rs8176318; G>T) with the risk of BC in the 3′-UTRs of the BRCA1 SNPs was investigated. The risk of BC was found to be significantly increased in individuals that had the GG or GT genotype when compared with those that had the TT genotype, in a Pakistani population. The TT genotype was observed to have decreased the risk of BC when compared to the GG genotype. The GG genotype had an OR of 1.557 and appeared to be the risk allele, while the TT allele had an OR of 0.47 and was found to provide protection against BC in the Pakistani population analyzed in the current study. Previous studies supported the observations made herein that variants within the miRNA binding sites of tumor suppressors or oncogenes play a pivotal role in the development of BC [13]. When comparing the frequencies of the 3′-UTRs of the BRCA1 G>T heterozygosity in this study group with those of other studies, it was found that the GT heterozygosity was generally comparable with Italy (TSI) at 50%; Chinese in Colorado (CHD) at 56%; Gujarati Indians in Houston, Texas (GIH) at 52%; European ancestry in Utah at %45; and 50% in a Saudi Arabian population [14]. Herein, the frequencies of the GG (31.33%), GT (52.0%), and TT (16.67%) genotypes in the patients were comparable to those of the GIH (29.5%, 52.3%, and 18.2%, respectively). Our G (57.3%) and T (42.6%) allele frequencies were comparable to those of the GIH (55.7% and 44.3%) and CHD (56.5% and 44.5%) [15]. Previously, a study of African-American women reported that the rs3092995 in the 3′-UTRs of the *BRCA1* increased the risk of BC [16]. In support of our findings, another study showed that the nrs8176318 GG genotype had a significantly lower expression level (of BRCA1 mRNA) than the GT and TT genotypes. Previous studies have supported our observations that variants within the miRNA binding sites of tumor suppressors or oncogenes play a role in BC development [13]. Other studies have reported that different alleles, rs12516 and rs8176318, increased the risk of BC [17]. The rs8176318 G/T variant was linked with increased risk of BC in studies across different populations worldwide [15]. The rs8176318 has also been reported to be a risk factor in African-American women, with an OR of 12.19 [18]. In contrast to our findings, some studies on variants inside the 3′-UTRs of the* BRCA1* have failed to show association with the risk of BC [19]. Similarly, in another contrasting study, the rs8176318 (T) variant in the* BRCA1 *has been shown to lower the gene expression and was associated with advancing the BC stage. According to another study, a 4-fold increase was observed in the shift to stage V of the disease [8]. That study is carried out on an Irish population, whereas the study herein was carried out on Asian population. Their study reported an increased risk of triple-negative BC patients. Our results reveal that the newly identified TT allele may be a new genetic marker for a decreased risk of sporadic BC. On the other hand, women with the GG allele are at an increased risk of BC. It has been shown that the mutant (T) allele reduces the BRCA1 expression in response to a decrease in the amount of estrogen when compared to the G allele, which showed that estrogen treatment dysregulated the T-allele expression, causing the down-regulation of the *BRCA1* [8]. The results of our study presented a variation in the noncoding region of the *BRCA1* that can influence its expression, increasing the risk of BC in Pakistani females. Evidence supporting the notion that susceptibility to BC is increased by variants inside the 3′-UTRs is on the rise [20]. The reason for the association of SNPs in the 3′-UTRs with increased risk of BC may be due to the fact that miRNAs bind to mRNA in the 3′-UTRs; thus, regulating the levels of mRNA and its expression [21]. In support of our findings, it was indicated by previous work that 3′-UTR variants act as genetic markers of the risk of cancer [22]. The major reason for discrepancies in our results with those of other studies was the diverse ethnic group (study population).

It was concluded that females with the GG genotype invariant (rs8176318; G>T) had a higher risk of developing BC, and women with the TT genotype in the 3′-UTRs of the* BRCA1 *had less chance of developing BC when compared to those with the GG genotype.

## Acknowledgment

The authors are extremely grateful to the Higher Education Commission of Pakistan for providing financial support under the Indigenous PhD scholarship Scheme to Mr. Mushtaq Ahmad (Ref. No. 213-59624-2BM2-131).
